# A comparison of single-channel and multi-channel RF transmit coil for SSFP cine imaging at 3 Tesla

**DOI:** 10.1186/1532-429X-13-S1-O10

**Published:** 2011-02-02

**Authors:** Shazia Hussain, Amedeo Chiribiri, Masaki Ishida, Geraint Morton, Andreas Wiethoff, Andreas Schuster, Eike Nagel

**Affiliations:** 1 NIHR Biomedical Research Centre- King's College London, London, UK; 2 Wellcome Trust and EPSRC Medical Engineering Centre- King's College London, London, UK; 3 King's College London, London, UK

## Introduction

On high field MRI scanners uniform radio frequency (RF) excitation over the entire field-of-view (FOV) is often challenging with single-channel RF transmit coils. This may cause a reduction of image quality (e.g. shadowing artifacts). This problem is most pronounced in sequences that heavily rely on a homogenous magnetic field, such as steady state free precession (SSFP) sequences. Multi-channel RF transmission (Tx) has been shown to significantly improve the RF (B1 field) uniformity in high-field MRI. More accurate knowledge of the local B1 field also allows to better predicting the local specific absorption rate (SAR), thereby allowing for shorter repetition times (TR). Overall, this may allow for an improvement in image quality as a consequence of reduced artefact

## Purpose

The purpose of this study is to evaluate whether the use of a multi-channel RF transmit coil can improve image quality of functional cine images compared to a conventional single-channel RF transmit coil

## Methods

Twelve subjects (eight patients and four healthy volunteers) were scanned at 3 Tesla (Achieva, Philips, Netherlands), acquiring SSFP cine in the short and long axis with and without Tx. Patients were randomized to either Tx or not Tx. Volunteers were scanned with both sequences. Typical sequence parameters for Tx /not Tx were: TR 2.9/3.5, TE 1.4/1.8, Flip angle 45/45, 30 acquired phases, matrix size 256x256). A blinded, expert reviewer assessed the image quality of the cine images and late gadolinium enhancement and scored image quality as follows: 1. Not diagnostic; 2. Poor (Diagnostic on most segments but not all); 3, Good (Diagnostic but not perfect); 4. Excellent image quality.

## Results

**Figure 1:**
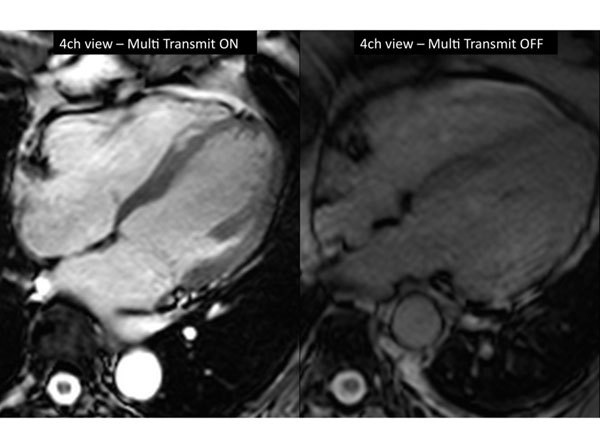
Comparison of a 4-chamber view SSFP cine with multitransmit ON and OFF.

Tx sequences offered a significantly higher image quality, both in patients and volunteers (see table 1). Figure 1 shows an example of images with and without the use of Tx.

**Table 1:** 

	Volunteers (n=4)		Patients (n=8)	
	*TX sequence*	*Non-TX sequence*	*TX sequence*	*Non-TX sequence*

**Average Score**	3.5	2.9	2.8	2.1
**St Dev**	0.9	0.9	0.8	1.1
**T-test**	0.014		0.030	

## Conclusion

Multi-transmit RF transmission allows for an improved SAR model with a significant reduction of TR and improved B1 homogeneity, leading to improved image quality of SSFP cine images. This will allow improved evaluation of patients with the SSFP sequence at 3T.

